# Implementation of non-linear mixed effects models defined by fractional differential equations

**DOI:** 10.1007/s10928-023-09851-1

**Published:** 2023-03-21

**Authors:** Christos Kaikousidis, Aristides Dokoumetzidis

**Affiliations:** Department of Pharmacy, National and Kapodistrian University of Athens, Panepistimiopolis, 15771 Athens, Greece

**Keywords:** Fractional differential equations, Anomalous kinetics, Numerical solver

## Abstract

Fractional differential equations (FDEs), i.e. differential equations with derivatives of non-integer order, can describe certain experimental datasets more accurately than classic models and have found application in pharmacokinetics (PKs), but wider applicability has been hindered by the lack of appropriate software. In the present work an extension of NONMEM software is introduced, as a FORTRAN subroutine, that allows the definition of nonlinear mixed effects (NLME) models with FDEs. The new subroutine can handle arbitrary user defined linear and nonlinear models with multiple equations, and multiple doses and can be integrated in NONMEM workflows seamlessly, working well with third party packages. The performance of the subroutine in parameter estimation exercises, with simple linear and nonlinear (Michaelis–Menten) fractional PK models has been evaluated by simulations and an application to a real clinical dataset of diazepam is presented. In the simulation study, model parameters were estimated for each of 100 simulated datasets for the two models. The relative mean bias (RMB) and relative root mean square error (RRMSE) were calculated in order to assess the bias and precision of the methodology. In all cases both RMB and RRMSE were below 20% showing high accuracy and precision for the estimates. For the diazepam application the fractional model that best described the drug kinetics was a one-compartment linear model which had similar performance, according to diagnostic plots and Visual Predictive Check, to a three-compartment classic model, but including four less parameters than the latter. To the best of our knowledge, it is the first attempt to use FDE systems in an NLME framework, so the approach could be of interest to other disciplines apart from PKs.

## Introduction

Fractional calculus is a mathematical branch investigating the properties of derivatives and integrals of non-integer orders and goes back to the Leibniz’s note in his letter to L’Hospital in which the meaning of the derivative of order one half is discussed [1]. Though for a long of time, fractional derivatives have existed only as theoretical mathematical objects, in the last few decades many authors have pointed out that mathematical models containing non-integer order derivatives and integrals can more adequately describe properties and phenomena that govern real materials e.g. polymers. It has also been pointed out that fractional differential equations (FDEs) that arise from the use of such non-integer derivatives, can be used to describe anomalous diffusion (i.e. diffusion not described by Fick’s law) and as a result, the anomalous kinetics that arise from it. In fact several experimental datasets have been described by fractional models better than by models of ordinary differential equations. One of the main features of FDE models, are the memory effects that arise by the fact that the value of a fractional derivative depends on a history of previous values and only on neighbouring ones [1].

In the field of pharmacokinetics (PKs), Dokoumetzidis and Macheras [2] introduced fractional calculus in 2009 to describe the PK of amiodarone that follows power law kinetics, and after that a few applications from the same and other authors followed, such as, to name a few, dealing with multicompartmental aspects of FDEs [3, 4], describing propofol PK [5], dealing with dose adjustment when powerlaw kinetics apply [6] and also extending the approach to pharmacodynamics, PDs [7]. Some applications have also appeared in neighbouring fields such as drug release kinetics [8] and skin drug absorption [9]. Recent applications of FDEs such as in the oral absorption of gentamicin [10] and bone remodeling [11] demonstrate that this is an active field of research. However, the number of fractional PK or PD models published is relatively limited, despite the fact that FDEs have attractive features and have the advantage that are extensions of classic ODEs which appear as special case when the order is set to 1. This should give fractional models important flexibility for empirical modelling even in cases where mechanistic description is not the main objective. One of the reasons for the limited uptake of fractional calculus in pharmacometrics literature is the lack of appropriate software: (a) efficient numerical solvers and (b) integration in parameter estimation software. A particular class of models of high importance in pharmacometrics is nonlinear mixed effects models (NLMEs) where variability components are also estimated for each parameter, typically by analysing longitudinal data (such as PK data) from many subjects and therefore characterising the interindividual variability of the parameters. For NLME models complex algorithms are used with various strategies for the calculation or approximation of the objective function and its minimization to the optimal parameter solution, and very rarely do scientists use homemade code for such a task. As a result, a few specialized software packages, mainly commercial and some open source, dominate all the NLME applications, with the leading one being NONMEM (Icon plc), which was the first one that appeared in the seventies and has continued to be the industry standard since then. It is therefore of particular importance to implement a NONMEM extension for FDEs that readily allows the description of fractional models.

In the present paper an extension of NONMEM is introduced that allows the definition of models including FDEs. The extension allows the user to try out FDEs in a model development workflow much like any other model using the same likelihood approximation and minimisation algorithms. Furthermore an implementation of an efficient numerical solver has been carried out, after extensive evaluation of various numerical solvers that appear in literature, a few of which are reviewed here too. The performance of the extension in parameter estimation exercises with linear and nonlinear fractional models has been evaluated by simulations and an application in a real clinical dataset of diazepam is presented. For completeness a brief introductory theoretical section on fractional calculus is also included.

## Fractional calculus

### Theory

The basis of fractional calculus is the definition of a fractional order of differentiation $\alpha$ -in our case $0< \alpha < 1$. This $\alpha$th derivative is defined through fractional integration and successive ordinary differentiation. Hence firstly a fractional integral formula is defined through the Riemann–Liouville (LR) integral [1]

1 $${}^{RL}_{0}I^{\alpha }_{t} f(t) ={}^{}_{0}D^{-\alpha }_{t} f(t)=\dfrac{1}{\Gamma (\alpha )} \int _{0}^{t} (t-\tau )^{\alpha -1} f(\tau )\ d\tau , \quad \alpha >0,$$

where $\Gamma$ is the gamma function. Here f is assumed such that the involved integral is well defined. The left-side subscript of the ${}^{RL}_{0}I^{\alpha }_{t}$ and ${}^{}_{0}D^{-\alpha }_{t}$ operators, denotes the lower end of the integration limits, which in this case has been assumed to be zero. Different bounds can be used but result in slightly different definitions and properties. Using ordinary differentiation of the above integral, the fractional derivative is defined through

2 $${}^{}_{0}D^{\alpha }_{t} f(t):={}^{}_{}D^{1}_{} \ {}^{}_{0}D^{\alpha -1}_{t} f(t)={}^{}_{}D^{1}_{} \ {}^{RL}_{0}I^{1-\alpha }_{t} f(t),$$

where ${}^{}_{}D^{1}_{}=\dfrac{d}{dt}$, the ordinary derivative. The calculations above yield the following definition for the LR fractional derivative [1]

3 $${}^{}_{0}D^{\alpha }_{t} f(t)=\dfrac{d}{dt}\left[ \dfrac{1}{\Gamma (\alpha -1)}\int _{0}^{t} \dfrac{f(\tau )}{(t-\tau )^{\alpha }} \ d\tau \right] , \quad \ 0< \alpha < 1.$$

This definition is a convolution between the function *f* and a power law function of time and is the source of the memory effects that arise through the process.

Though widely known, the LR definition of fractional derivatives is not the most useful one when it comes to real applications. That is because when used in differential equations, for $t=0$ a fractional integral of the function *f* is involved in the initial condition which is not easy to interpret physically. That is why another definition is mostly used when dealing with FDEs, and that is the Caputo derivative which is defined as

4 $${{}^{C}_{0}D^{\alpha }_{t}f(t):=\dfrac{1}{\Gamma (1-\alpha )}\int _{0}^{t} \dfrac{f^{\prime }(\tau ) \ d\tau }{(t-\tau )^{\alpha }}}, \quad \ (0<\alpha <1),$$

where the *C* in the upper index indicates the Caputo definition of the fractional derivative. With this definition, the initial value problems for fractional problems can be written in the familiar form

5 $${}^{C}_{0}D^{\alpha }_{t}y(t)=f(t,y), \quad y(0)=y_{0}.$$

Another advantage this definition has is that it carries properties that are consistent to the ordinary derivative such as that the fractional derivative of a constant is zero ${}^{C}_{0}D^{\alpha }_{t} \ C=0$ which is not the case for LR. For these reasons in the rest of this paper, the fractional derivatives used are assumed to be Caputo derivatives.

A simple example of an FDE from PKs is the classic one-compartment model with first order elimination

6 $$\dfrac{dA}{dt}=-k_{e}A, \quad A(0)=A_{0}.$$

The fractional equivalent of this equation is

7 $${}^{C}_{0}D^{\alpha }_{t}A(t)=-k_{e} A(t), \quad A(0)=A_{0}$$

and its solution can be expressed analytically through the Mittag–Leffler function [1]

8 $$A(t)=A_{0} E_{\alpha }(-k_{e} t^{\alpha }),$$

where

9 $${E_{\alpha }(x)= \sum _{k=1}^{\infty } \dfrac{x^{k}}{\Gamma ({\alpha }\cdot k+1)}}.$$

This is a generalised form of the exponential function since for $\alpha =1$ we see that $E_{1}(x)=exp(x)$. This means that the fractional version of the simple PK model collapses to the ordinary one for $\alpha =1$, $A(t)=A_{0} e^{-k_{e} t}$. In [12] it is shown that (8) behaves as a stretched exponential for small times i.e. $\sim exp(-k_{e} t^{\alpha })$ while for larger times behaves like a power law. In general the solution of this fractional equation can describe anomalous processes or drug release in heterogeneous media [12].

In actual applications few problems of the form of (5) have a closed-form analytical solution. In fact even (7) which does have an analytical solution, still needs to be computed numerically in order to approximate the Mittag–Leffler function efficiently, since the analytical definition involves an infinite power series which is more difficult to compute. Hence the necessity of numerical methods which can approximate the trajectories of such fractional systems arises naturally. Numerical solvers for FDEs must be efficient both in terms of accuracy as well as speed. In the next section a few of these methods are presented with emphasis on the Grünwald–Letnikov (GL) scheme [13] that was the one chosen for the implementation in NONMEM and was used to produce the results presented in this paper.

### Numerical methods

#### The Laplace transform

A function *f*(*t*) is of exponential order *a* when we can find positive constants *M* and *T* such that $e^{-at} |f(t) |\le M$ for all $t>T$ so *f*(*t*) does not grow faster than an exponential function for $t\rightarrow {\infty }$. The Laplace transform for such a function is defined [1]

10 $$F(s)={\mathcal {L}}\left[ f(t);s\right] :=\int _{0}^{\infty }e^{-st}f(t) dt.$$

The inverse Laplace transform can also be defined, though it is not possible to write it down in most cases. The inverse transform gives the original function

11 $${\mathcal {L}}^{-1}\left[ F(s);t\right] :=\int _{c-i\infty }^{c+i\infty }e^{st}F(s)ds=f(t), \quad c=Re(s)>c_{0},$$

where *c* is the path of integration. In classic calculus, the Laplace transform is a useful technique for solving linear ordinary differential equations. In the Laplace domain one can transform ODEs to algebraic expressions which are much easier to solve, and then go back to the time domain by performing the inverse Laplace transform. Fractional linear differential equations can also be written in the Laplace domain easily since—like ordinary derivatives—fractional derivatives can be transformed in the Laplace domain. For $0< \alpha \le 1$ it can be shown that [1]

12 $${\mathcal {L}}\left[ {}^{C}_{0}D^{\alpha }_{t} f(t);s\right] =s^{\alpha} F(s)- s^{\alpha -1}f(0).$$

A simple example of an FDE that can be solved using the Laplace transform is found in [3]

13 $${}^{C}_{0}D^{1/2}_{t}y(t)=-y(t)$$

with $y(0)=1$. Using (12) it can be written as

14 $$s^{1/2} Y(s)-s^{-1/2}y(0)=-Y(s),$$

y where *Y*(*s*) is the Laplace transform of *y*(*t*). Using standard algebra it gives the following result in the Laplace domain

15 $$Y(s)=\dfrac{1}{s+\sqrt{s}}.$$

The final solution can now be obtained by performing an inverse Laplace transform. For this simple example, there are expressions that can be found in tables with inverse Laplace transform formulas such as [1] which yield the known result

16 $$y(t)=E_{1/2}(-t^{1/2}).$$

Unlike the previous example, solving (11) explicitly for the system of variables and obtaining an analytical solution for *Q*(*s*) in the time domain, is generally difficult—if not impossible. To address this problem algorithms to carry out numerical inverse Laplace transform (NILT) exist. Several such algorithms have been developed such as [14] which is based on the Euler algorithm [15]. Another popular choice is the Talbot method [16] where the path of integration is chosen to be a special cotangent contour [17]. These algorithms are a useful tool for solving linear FDEs and have been used successfully in fractional PKs estimation problems [3], however they are typically very demanding computationally. This makes them unattractive for estimation problems where the speed of the algorithm is of paramount importance.

#### Fractional linear multi-step methods

An alternative to the various NILT methods that provides more efficiency as well as universality, is the family of methods known as fractional linear multi-step methods (FLMMs). These are extensions of the classic Linear Multi-Step methods used for numerically solving classic ODEs. The name multi-step stems from the fact that they use the solution at several steps previous to the current step for which the solution is computed. The idea behind these methods is that an initial value problem

17 $$y'(t)=f(t,y) \quad \ y(0)=y_{0}$$

can be written as

18 $$y(t)=y_{0}+\int _{t_{0}}^{t}f(t,y)dt$$

which can be approximated by [13]

19 $$\sum _{j=0}^{k} \rho _{j} y_{n-j}=h\sum _{j=0}^{k}\sigma _{j} f(t_{n-j},y_{n-j}),$$

where $\rho (z)=\rho _{0} z^{k}+\rho _{1} z^{k-1}+ \cdots +\rho _{k}$ and $\sigma (z)=\sigma _{0} z^{k}+\sigma _{1} z^{k-1}+ \cdots +\sigma _{k}$ are the first and second characteristic polynomials. The fraction of these two polynomials give the generating function of the LMM

20 $$\delta (\xi )=\dfrac{\rho (1/\xi )}{\sigma (1/\xi )},$$

where meaning of $\delta (\xi )$ is that the weights $\omega _{n}$ can be written as the coefficients of the power series of the generating function. Depending on whether $\rho _{0} \ne 0$ and $\sigma _{0} \ne 0$, the method is either explicit [$y_{n}$ appears only on the left side of (19)] or implicit ($y_{n}$ appears on both sides). Finally this can be reformulated in terms of convolution quadrature formulas in:

21 $$y_{n}=h\sum _{j=0}^{n}\omega _{n-j}f(t_{j}).$$

This method is a special case of a general methodology used for solving problems of type (18) or otherwise known as Volterra integral equation of the second kind [18]. The extension to FDEs of the general LMMs is based on the fact that the general problem (5) can also be written as a Volterra integral equation simply by the fractional integral operator (${}^{}_{0}I^{\alpha }_{t}$) on both sides and obtaining

22 $$y_{n}=y_{0}+\frac{1}{\Gamma (\alpha )}\sum _{j=1}^{n}\int _{t_{j-1}}^{t_{j}}(t_{n}-\tau )^{\alpha -1}f(\tau ,y(\tau ))d\tau .$$

The details on deriving the fractional equivalent of (21) can be found in [19]. There, the final formula for the FLMMs is derived

23 $$y_{n}=y_{0}+h^{\alpha} \sum _{0}^{s}w_{n,j}f(t_{j},y_{j})+h^{\alpha} \sum _{0}^{n}\omega ^{(\alpha )}_{n-j}f(t_{J},y_{j}), \quad \alpha \le 1$$

and $w_{n,j}$ are correction terms which maintain the order of convergence when *f* is non-smooth [19]

In this work, the method that was used for the numerical solution of the FDEs was the so called GL scheme based on (23). It is basically a backwards differentiation formula of first order (BDF1). The correction terms are omitted and using the classic BDF1 where the generating function takes the form $\delta (\xi )=1-\xi$, it can be shown [18] that the $\omega$ coefficients are those of a binomial series. Hence

24 $$\omega _{n}^{(\alpha )}=(-1)^{n}\left( {\begin{array}{c}-\alpha \\ n\end{array}}\right) =(-1)^{n}\dfrac{\Gamma (1-\alpha )}{\Gamma (n+1)\Gamma (-\alpha -n+1)}$$

and so the formula for $y_{j}$ is

25 $$y_{n}=y_{0}+h^{\alpha} \sum _{j=0}^{n}(-1)^{n-j}\left( {\begin{array}{c}-\alpha \\ n-j\end{array}}\right) f(t_{j},y_{j}), \quad \alpha \le 1,$$

where $y_{0}=y(0)$. Since this method is implicit there is need for an approximation of $y_{n}$ to be used in $f(t_{j},y_{j})$ for $j=n$. In this work, an iterative Newton–Raphson method was used for this purpose. Given an initial approximation for $y_{n}$—let this be $y_{n}^{(0)}$— the method can approximate $y_{n}$ using the following relation

26 $$\begin{aligned}&y_{n}^{(k)}=y_{n}^{(k-1)}-\left[ 1-h^{\alpha} f'(t_{n},y_{n}^{(k-1)})\right] ^{-1} \\&\quad \times \left[ y_{n}^{(k-1)} -h^{\alpha} f(t_{n},y_{n}^{(k-1)})-\sum _{j=1}^{n-1}\omega _{n-j}^{(\alpha )} f(t_{j},y_{j})-y_{0} \right] ,\end{aligned}$$

where $k\in {\mathbb {N}}$ is the number of iterations. This method is highly popular because of its quadratic convergence [20]. Since its convergence properties are local, a good initial estimate $y_{n}^{0}$ is required for the method to perform well. A good approximation for it is usually $y_{n}^{(0)}=y_{n-1}$. In rapidly changing solutions (i.e. non-smooth functions) a smaller step is required for this approximation to provide accurate results.

#### Impulsive fractional differential equation

A particular class of problems that is of interest in PK and PK/PD applications, are the impulsive differential equations which are described by

27 $$\begin{aligned}&{}^{C}_{0}D^{a}_{t} y(t)=f(t,y(t)), \quad t\in J':=J \setminus \left[ t_{1},\dots ,t_{m}\right],  \, J:=[0,T] \\&y(t_{k}^+)=y(t_{k}^-)+u_{k}, \quad k=1,2,\dots ,m, \quad y(0)=y_0 \end{aligned}$$

which means that for every time point $t_{k}$ there is an impulse $u_{k}$ that mainly describes physical phenomena that have a sudden change in their states, i.e. an additional dose, where the total number of doses is *m*. These types of equations are therefore able to describe multiple dose kinetics. In the classic case ($\alpha =1$), the method of solving these problems is trivial, since one can simply solve ODEs for time intervals between doses and then for $t_{k}$ restart the solver with initial conditions containing the extra dose. For $(a< 1)$ that is not the case since as mentioned, each $y_{i}$ is depends on the solution of each previous time point, therefore restarting the solver for each dose, would result in loss of that information. For the numerical solution of this class of problems, we follow [21] that suggests that the solution of (27) is

28 $$\begin{aligned}&y(t)=y_{0}+\sum _{i=1}^{k}u_{k}+\dfrac{1}{\Gamma (a)}\int _{0}^{t} (t-s)^{a-1}f(s,y(s)) \ ds, \\&\ \ \text {for} \quad t\in (t_{k},t_{k+1}). \end{aligned}$$

This problem is identical in its numerical solution to (22) but for the addition of the summation term of the doses $u_{k}$ which is implemented in the existing algorithm with a simple *if* statement which adds each dose to each $y_{n}$ for $t_{n}>t_{k}$.

## Methods

### Numerical solver for fractional differential equations

The GL scheme was the method chosen for the solution of the FDEs that described the models tested in NONMEM. The algorithm was picked for its efficiency in terms of speed and accuracy compared to other algorithms. More specifically, the Adams–Bashforth–Moulton predictor–corrector (ABMPC) method [12] which was also tested, proved both slower and less accurate for small *t* than the GL algorithm. The nature of the ABMPC algorithm is such that more computations accumulate in the process thus increasing computational time. Also for small times there were terms that needed small steps *h* in order to converge which was time costing by itself. However it is worth mentioning that this algorithm was robust and efforts have been made to reduce computational costs [22], making it a possible option for use on more complex problems.

In order to validate the algorithm upon which the numerical solver was based, various tests were performed. The rationale was to compare the numerical solution preferably to an analytical solution, so for cases where an analytical solution was not available such as non-linear models comparison for the classic case of $\alpha =1$ was used. First, the numerical solution was computed for a linear fractional PK model like (7) and compared to the analytical solution given by (8). In order to verify that the algorithm gives reliable results for non-linear models as well, it was tested on a fractional Michaelis–Menten equivalent model (29), by setting $\alpha =1$ and comparing the results to the classic Michaelis–Menten solution, since neither an analytical solution nor a NILT solution is available for this case. The capability of the algorithm to handle multiple FDEs were tested by simulating a classic two-compartment PK model with $\alpha =1$ and compared to the analytical solution. Finally the capability of the algorithm to handle multiple doses was tested with the linear fractional model of Eq. (7) compared to the superposition of multiple instances of the analytical solution of Eq. (9). Indeed, in linear FDEs, superposition principle holds, but the multiple dose problem in the nonlinear case, is nontrivial due the memory effects. All the above mentioned algorithms were developed and tested in MATLAB before final implementation in FORTRAN. The GL algorithm was optimized for speed and accuracy.

### NONMEM implementation and interface

The numerical routine for the solution of FDEs was developed and tested in MATLAB, and ported to FORTRAN to make it available as a subroutine in NONMEM. It supports linear and nonlinear models, with multiple FDEs, as well as multiple doses. The implementation of the fractional models in NONMEM is done using $PRED control record in the control file in order to provide a user supplied subroutine for the solution of the model equations. These are not defined in the control file like in the $DES case but in the subroutine itself. The subroutine FDEGL(...) can be called anywhere in $PRED using a brief verbatim code “CALL FDEGL(VECTRA, VECTRB, TIME, ff, AMT, int(Np), int(Ns), int(Nd)), given that the all the arguments used, are defined. The vector names VECTRA, VECTRB are used in order to be compliant with what the NONMEM abbreviated code supports. Arguments are: All model parameters including the order of the fractional derivative $$\alpha$$ are passed in VECTRA; VECTRB stores the initial values of the dependent variables; TIME is the value of the NONMEM data record for which the solution is to be computed; AMT is the data record needed for each dose, as usually used in NONMEM; Np the number of the parameters, Ns is the number of steps for the solver and Nd the number of FDEs. Once called, it returns the solution of the equation, ff, for each time point requested by NONMEM (i.e. the TIME value for each data record).

The FORTRAN subroutine consists of two parts: the main solver algorithm and the definition of the FDE. The first part is fixed and is not supposed to be changed by the user. The second part of the subroutine is the definition of the problem and consists of two FORTRAN functions.

In the first FORTRAN function, FFUN, the user has to input right hand side of the FDE (5) exactly as with all ODE solving software. In the second FORTRAN function, FFUNJ, the user inputs the derivative $\partial f(t,y)/ \partial y$ in the same manner. For example, in the simple system of (7), the first function is FFUN=-ke*y, while the second function is FFUNJ=-ke, which is the Jacobian of the right hand side of (7).

This—apart from the number of steps of the solution—is the only user input in the process, which makes the use of the subroutine easy and familiar thus requiring little or no knowledge of fractional calculus on behalf of the user. Also note that doses are handled naturally with in the data file in the AMT data item, as usual.

The FORTRAN subroutine FDEGL together with a template NONMEM control stream, as well as user instructions, can be found in a GitHub page (https://github.com/PMXathens/FDE4NONMEM). All work in this paper was carried out using NONMEM version 7.4 compiled with gfortran version 4.6.0.

### Evaluation by simulation study

Several tests on single patient runs were performed to make sure the algorithm was called properly by NM-TRAN. Afterwards one hundred data sets were simulated for each of the two models studied and estimation of the population parameters was carried out for each one of them in order to calculate the bias and precision of the methodology, by the relative mean bias (RMB) and relative root mean square error (RRMSE), respectively. In both models—linear and non-linear—a population of 24 subjects was simulated for $t=40$ h and both fixed and random effects were considered. The batch estimation process was achieved using the Bootstrap tool of Perl Speaks NONMEM (PSN) with data sets simulated in MATLAB. Namely, the datasets of the simulation study prepared in MATLAB, were placed in the bootstrap directory of a PSN bootstrap run, as bootstrap datasets and then the bootstrap command was run to execute them.

### Linear model

The linear model is given by the expression (7) with an additional parameter volume of distribution, *V*, as the proportionality constant between the drug amount *A* and the measured concentration *C*, as $C(t)=A(t)/V$. Variability was assumed in $k_{e}$ and *V*, whereas $\alpha$ was kept the same for all subjects. A log-normal distribution was considered for the two random variables and a proportional error model for the residual variability. More specifically, the $\theta$ values were: $\theta _{k_{e}}=0.5\ h^{-\alpha }$ , $\theta _{V}=6.5$ L and $\theta _{\alpha} =0.5$ while for the random distributions: $\omega _{k_{e}}=0.25$, $\omega _{V}=0.25$ for the interindividual variabilities and $\sigma =0.1$ for the proportional residual error and a dose of 9 mg. For the estimation process of each data set, the stochastic approximation expectation maximization (SAEM) method with INTERACTION was used since it proved the most robust of all other estimation methods for this problem, while the standard errors were estimated by the importance sampling (IMP) method.

### Non-linear model

As a second case study, a non linear model was chosen in order to show that the methods can be used in a wider category of problems. A fractional analog of Michaelis–Menten kinetic was used, described by the following equation

29 $${}^{C}_{0}D^{\alpha }_{t}A(t)=-\dfrac{V_{max}C(t)}{K_{m}+C(t)}$$

and $A(0)=dose$, $C(t)=A(t)/V$, where $K_{m}$ and $V_{max}$ are the Michaelis–Menten constant and the maximum rate of elimination, respectively. In the population study, log-normal variability was considered in $V_{max},\ K_{m}$ and *V* whereas $\alpha$ was kept the same for all patients. The simulation parameters were: $\theta _{V_{max}}=3.5 \ {\text {mg}}/h^{\alpha}$, $\theta _{K_{m}}=0.6$ mg/L, $\theta _{V}=2$ L, $\theta _{\alpha} =0.9$ with $\omega _{V_{max}}=0.25$, $\omega _{V}=0.25$, $\omega _{K_{m}}=0.25$ and a dose of 9 mg. Once again a proportional error model was used with $\sigma =0.1$ and the SAEM method with INTERACTION was chosen for the estimation process.

### Diazepam data analysis

For the application on a real data set, Diazepam data from [23] were used. The data set used consisted of 12 patients for $t=72$ h for an iv dose of 5–10 mg. A fractional one-compartment model was used for the fitting process, initially using three parameters: volume of distribution (V), elimination rate ($k_{e}$) and the order of the fractional derivative ($\alpha$). For comparison purposes, classic (non-fractional) 1, 2 and 3-compartment models with linear elimination were also fitted to the data. The comparison of the fits was done using diagnostic plots such as predicted vs observed and residuals vs time as well as Visual Predictive Check (VPC) plots using PSN.

## Results

### Numerical results

**Fig. 1:**
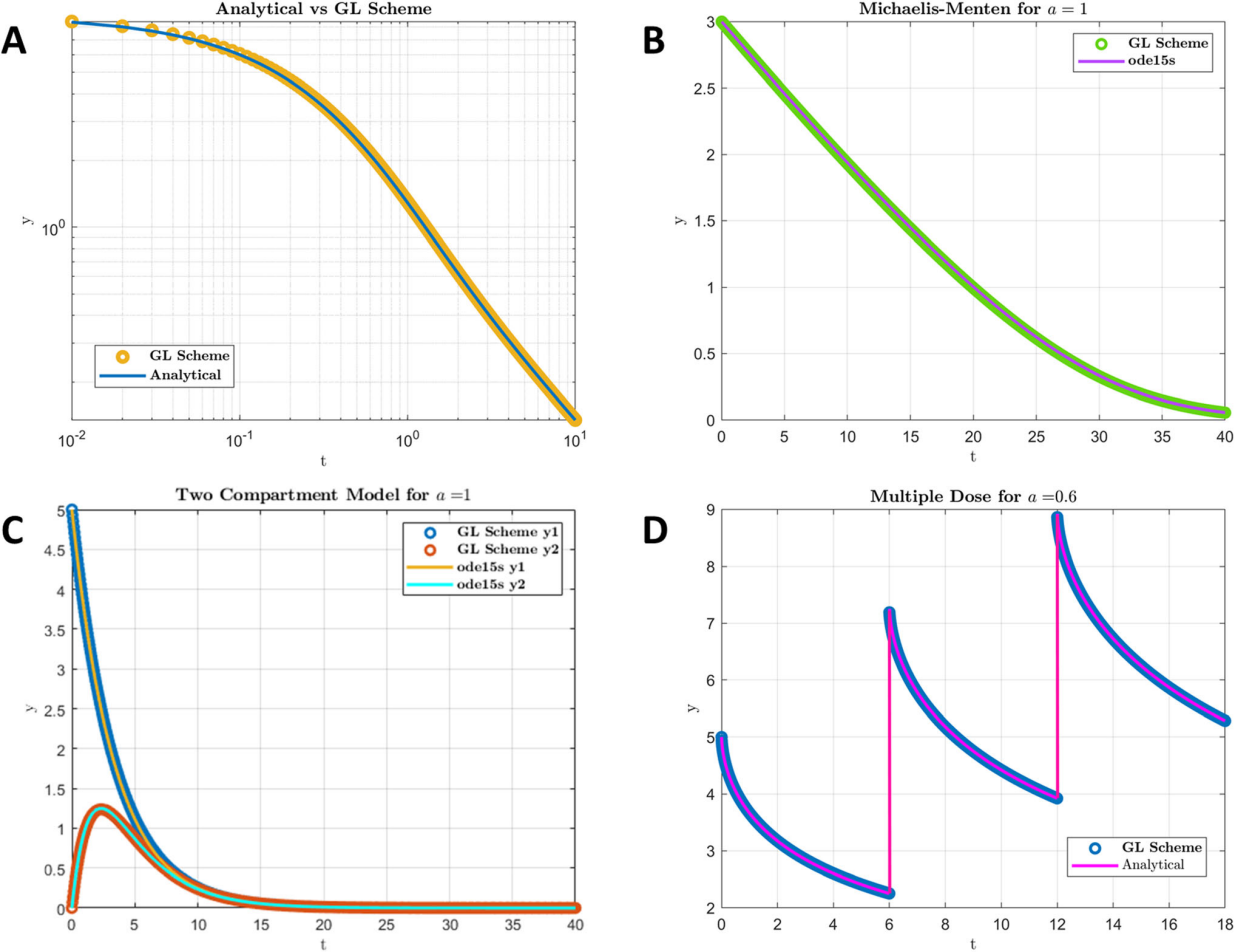
Typical profiles generated with GL scheme compared to various alternative methods. **A** GL scheme vs. Analytical ML function for the linear model. **B** GL scheme vs. MATLAB ode15s for classic (non-fractional) Michaelis–Menten model. **C** GL scheme vs. MATLAB ode15s for classic (non-fractional) two-compartment model. **D** GL scheme for multiple dose vs. superposition of analytical ML functions

In order to test the numerical solvers used for the solution of FDEs the GL scheme was compared to known methods. The results are shown in Fig. 1 with the compared solutions completely overlapping, demonstrating that the GL method is successful in solving both the linear and non-linear problems, while it can handle successfully multiple equations and multiple doses. Other than its accuracy, the method was efficient in terms of speed. More specifically, for the linear example of (7), the numerical solution was compared to the analytical solution (8) (Fig. 1A), while for the non-linear equation (29) a comparison was made for $\alpha =1$ i.e. the classic Michaelis–Menten equation, using a typical MATLAB solver (*ode*15*s*) (Fig. 1B). The multi-compartment case was tested also for the classic $\alpha =1$ against the solution with the same MATLAB solver (Fig. 1C). The main aspect tested in the two latter cases is the ability of the algorithm to give accurate results and it is thought that since in the classic case the GL method gives correct results, it is unlikely that it could produce a wrong solution when $\alpha <1$. In the last case the multiple dose scenario was tested for the linear model for $\alpha =0.6$, against the superposition of the respective analytical solution. While the superposition holds in linear FDEs and is a good approach to handle multiple doses, in the nonlinear case multiple doses need to be handled within the algorithm, while restarting the solution using as initial values the final point of the previous dose session, which is the common practice for ODEs, is not feasible in FDEs due to the memory effects and the presence of history. Figure 1D shows that multiple doses can be handed well within the GL solver for the fractional linear case and there is no reason to believe that this could change for the non-linear case, despite the fact that there is no analytical solution for the latter case to test it against.

### Simulation study

The NONMEM implementation was evaluated by a simulation/estimation study for the two models (7) and (29). The first is the simplest PK relationship, the iv bolus with linear elimination, which in its fractional version can account for various diffusion processes that are anomalous or of slower diffusion in the deeper tissues. The second model is the fractional version of a typical non-linear Michaelis–Menten model which is usually used to describe saturable PK processes. While its physical meaning is unclear at the moment, here it is used as a typical non-linear model to test the performance of the method.

Parameters were estimated for each of the one hundred simulated data sets for the two models. The RMB and RRMSE were calculated using the results from each estimation in order to assess the bias and precision of the methodology. The results for the linear and non-linear models are shown in Tables 1 and 2, respectively. For the linear model, RMB was below or near 1% while RRMSE below 10% for the THETAs and in the region of 15% for the OMEGAs and the SIGMA. In the non-linear model more elevated values for $V_{max}$ and $K_{m}$ were obtained for RMB, 5% and 11%, respectively, while for RRMSE the highest value was obtained for $K_{m}$ and was 19%. Overall, the results show high accuracy and precision for the estimates. Also the expected values for each parameter, i.e. the mean of the one hundred estimates, are very close to the simulated values, all of which indicate a good performance in the simulation study.

The performance of NONMEM while using the user function for the solution of the fractional equations, was satisfying since it did not produce unusual errors and warnings while the speed of the optimization was found to be relative to the speed of the solver.

**Table 1:** Results of the simulation study for the Linear Model

	$\theta _{k_{e}} \ (h^{-\alpha })$	$\theta _{V}$ (L)	$\theta _{\alpha}$	$\omega _{k_{e}}$	$\omega _{V}$	$\sigma$
RRMSE ($\%$)	9.14	0.95	2.72	16.53	12.03	14.62
RMB ($\%$)	$-1.017$	0.322	0.393	0.041	$-0.611$	$-0.036$
Sim. values	0.5	6.5	0.5	0.25	0.25	0.1
Exp. values	0.506	6.251	0.501	0.259	0.25	0.099

For each model, all 100 estimation runs were successful and all results were used for the calculation of RMB and RRMSE

**Table 2:** Results of the simulation study for the Non-Linear Model

	$\theta _{V_{max}}$ (${\text {mg}}/{h^{\alpha} }$)	$\theta _{K_{m}}$ (${\text {mg}}/{h^{\alpha} }$)	$\theta _{V}$	$\theta _{\alpha}$	$\omega _{V_{max}}$	$\omega _{K_{m}}$	$\omega _{V}$	$\sigma$
RRMSE ($\%$)	2.94	19.13	2.82	0.7	7.2	11.11	8.21	12.06
RMB ($\%$)	$-4.98$	$-11.03$	$-2.36$	$-0.62$	$-0.63$	$-1.54$	0.12	$-0.12$
Sim. values	3.5	0.6	2	0.9	0.25	0.25	0.25	0.1
Exp. values	3.422	0.55	1.998	0.893	0.249	0.244	0.25	0.099

For each model, all 100 estimation runs were successful and all results were used for the calculation of RMB and RRMSE

### Diazepam data analysis

The fractional model that best described the drug kinetics was a one-compartment linear model with an elimination rate $k_{e}$ and fractional order $\alpha$, i.e. (7). The volume of distribution V was not estimated but set to 1 since there was an identifiability issue. This is to be expected since, as already mentioned, the analytical solution of this equation is a Mittag–Leffler function given by (9). This function has two main trends: For small values of *t* it has the form of a stretched exponential function, while for large values of *t* it has the form of a power law. The analytical form of this power law function is given by the following expression [24]

30 $$y(t)=\dfrac{t^{-\alpha }}{\Gamma (1-\alpha )}\frac{V}{k_{e}}.$$

For the case of Diazepam, data were available for 72 h, while for initial times there was limited information, hence the solution was required for large times, following (30). This means that we expect only the ratio $V/k_{e}$ to be estimated properly since the solution is the same for any combination of *V* and $k_{e}$ that results in the same ratio. Thus in NONMEM only a $k_{e}$ parameter was estimated and the volume of distribution was arbitrarily set to 1.

**Fig. 2:**
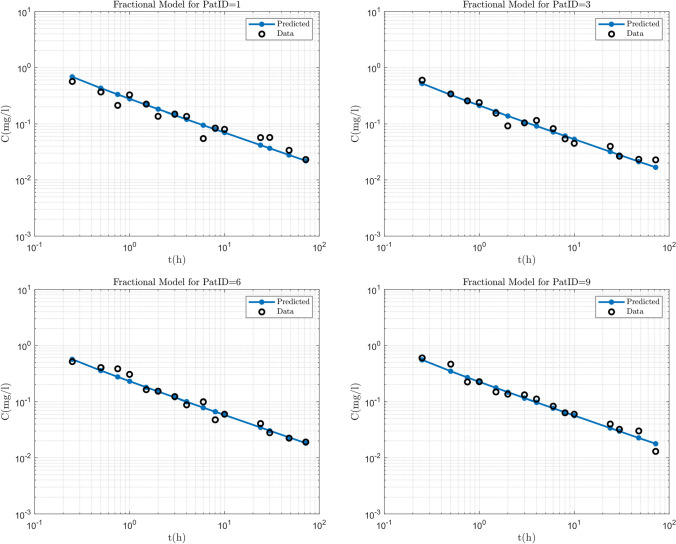
Individual fits for 4 out of the 12 patients, showing the power law behaviour of the data

**Fig. 3:**
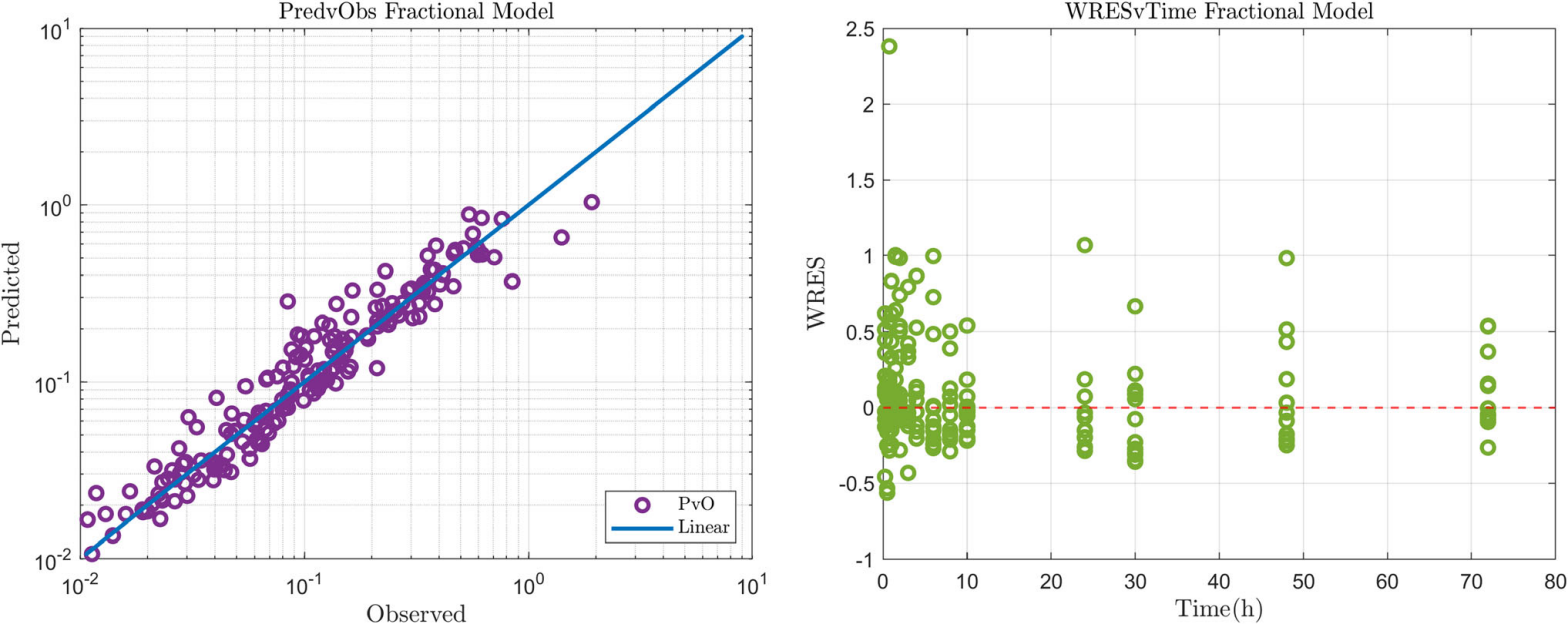
Predicted vs. Observed (left) and Residual versus Time (right) graphs

The estimation process was performed in NONMEM while the most successful method was SAEM with INTERACTION. Standard errors were calculated in NONMEM by the IMP method. The results of the NLME model constructed to fit the Diazepam data, were inspected visually and with the use of diagnostic plots. Predicted vs. Observed, Residuals vs. Time and VPC graphs were used to determine the goodness of fit as well as the ability of the model to predict the data. Some individual fits obtained by NONMEM can be seen in Fig. 2. The results are presented in log–log plots in order to assess the fit more accurately for the small concentration values. As shown by the plots, the Diazepam data follow a power law shape. The predicted versus observed and residual versus time graphs are presented in Fig. 3 showing all 12 patients together with the identity line and show good agreement between the predictions and the observations. The residual versus time plot shows that the points are randomly distributed evenly around the *x* axis which provides evidence that the estimator is not biased and also that the predicted points follow the same trend as the observed points.

**Table 3:** Parameter estimates for the diazepam data analysis

	$\theta _{k_{e}}$	$\theta _{\alpha}$	$\omega _{k_{e}}$	$\sigma$
Estimated values	17.1	0.54	0.1	0.083
Standard errors	2.04	0.017	0.0512	0.0084
CV ($\%$)	11.92	3.15	51.2	10.1

In Table 3 together the parameter estimates, the standard errors of the estimates are shown, which take small values indicating that the estimator is precise.

**Fig. 4:**
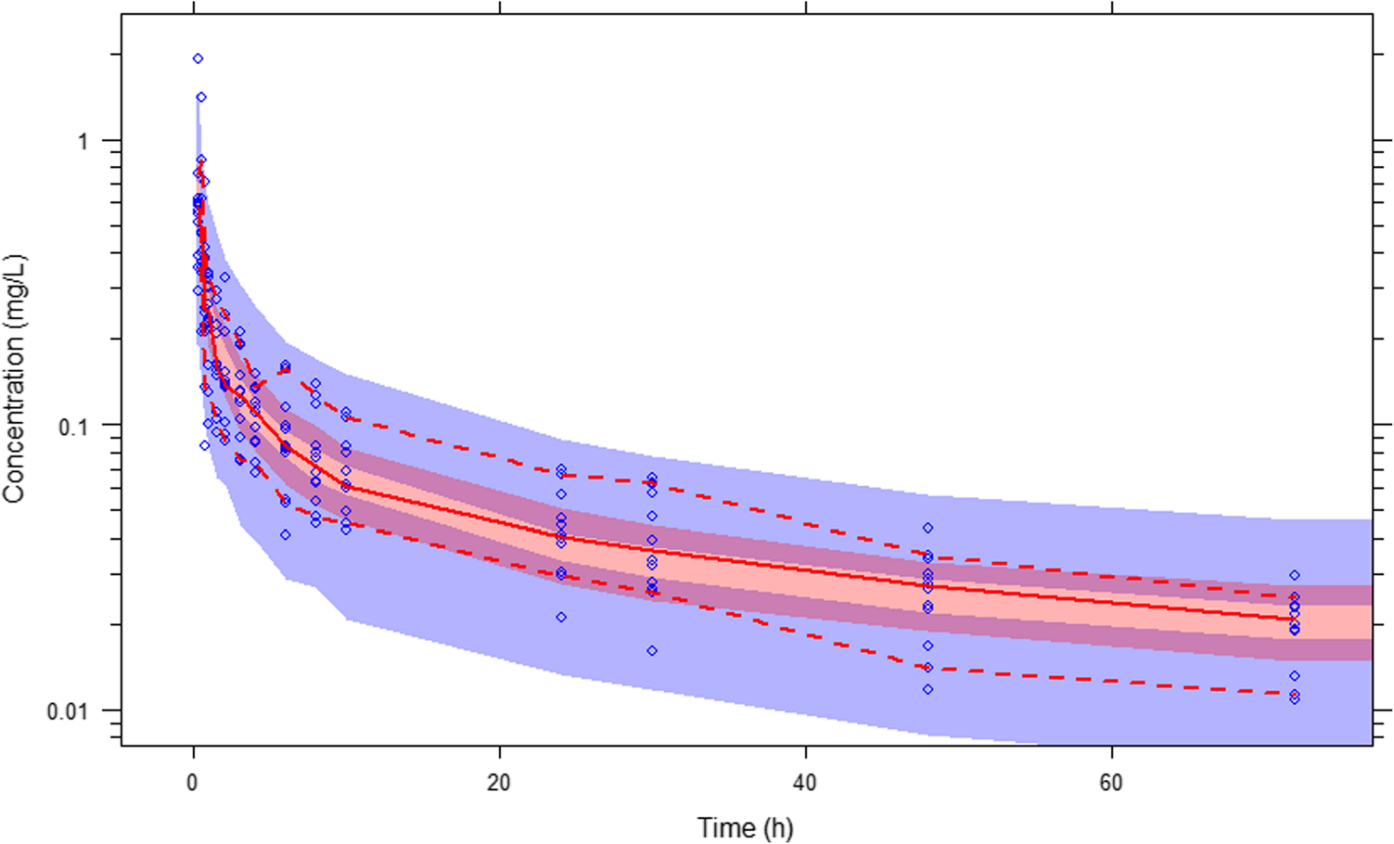
VPC for Fractional model

Finally the ability of the model to predict the data was evaluated with the use of a VPC, which also tests whether misspecification is present in the structural, variability or error models. For this procedure, 500 data sets of the same format as the observed data set were simulated and 95% prediction intervals around each of the 5th, 50th, and 95th percentiles of the simulated data sets were plotted together with the corresponding percentiles of the observed data. The results appear in Fig. 4 and show that the observed percentiles fall within the prediction intervals of the simulated data. The VPC plot was generated in PSN which demonstrates that companion third party software packages such as PSN work well with the present FDE NONMEM extension, without modifications.

**Fig. 5:**
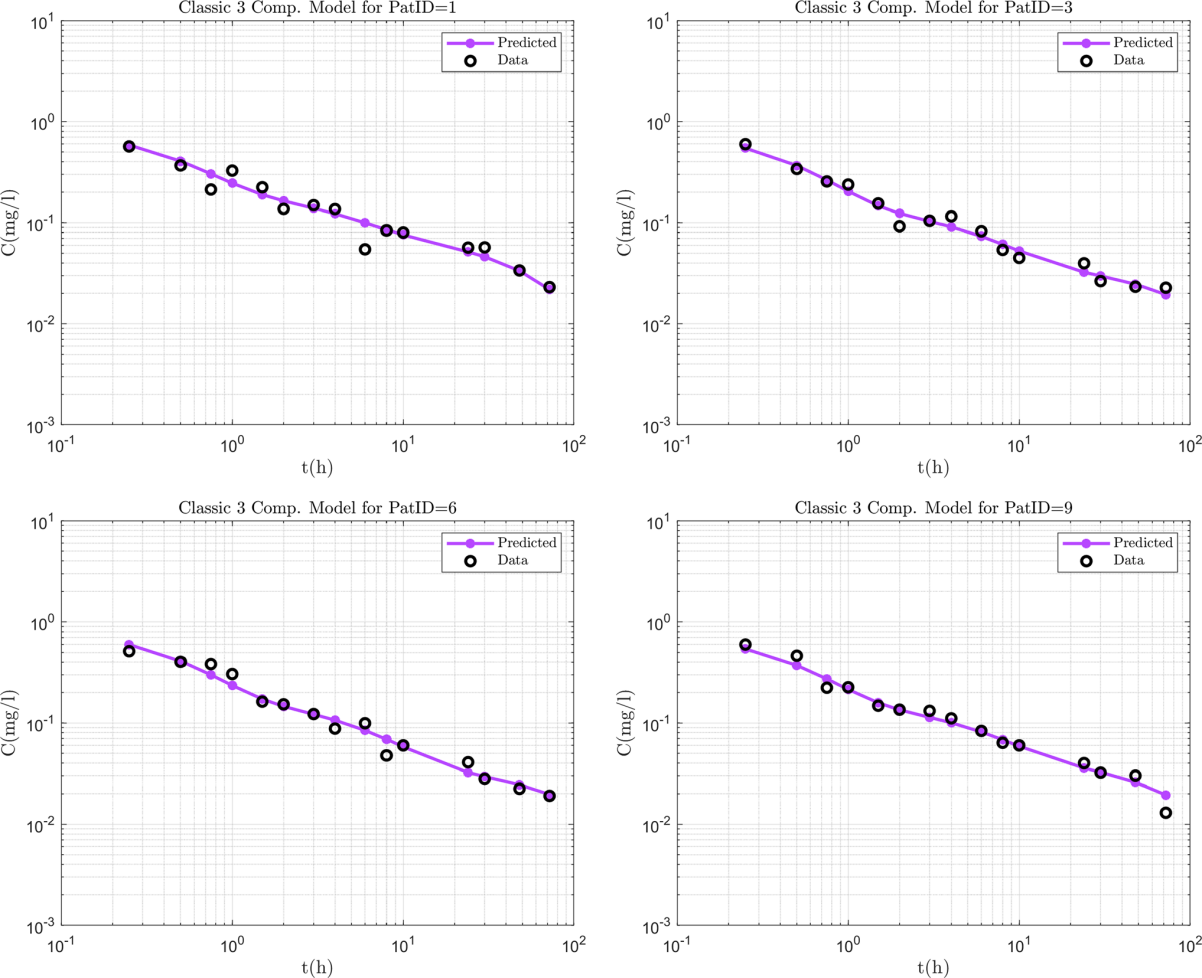
Individual fits for 4 out of the 12 patients, for a classic 3 compartment model with absorption for comparison

The fractional model was also compared to a classic compartment model. An optimization was performed using the same data set for Diazepam with a classic three-compartment model with iv bolus administration. The fit for the same patients as for the fractional model is presented for the classic case in Fig. 5. The fit with the three-compartment model is comparable to the fractional model but using six parameters as opposed to only two of the fractional model.

## Discussion

The main deliverable of the present work is a general purpose subroutine, written in FORTRAN, which works as a plugin to NONMEM and allows the definition of arbitrary, user-defined, linear and nonlinear FDE models. The actual numerical algorithm used for the FDE solver is based on the GL method [19], but has been modified to suit our needs. Furthermore the final choice of the numerical method is a result of an extensive survey and testing of many different algorithms in terms of performance and flexibility. To evaluate the approach, we presented a couple of simulation–estimation exercises for linear and nonlinear systems, respectively, and an application to a real PK clinical dataset.

Although successful in this study, this is a first approach of the use of fractional models in population analysis and the method still needs to be tested on more complex problems, where stiff differential equations or larger data sets appear. Also the method has limitations. At the moment only IV bolus dosing is supported, with multiple doses too. Other routes of administration or infusion would need adding to the model depot compartments which is not trivial. Also at the moment only FDEs of the form of Eq. (5) are supported, i.e. with the fractional derivative on the left hand side. More general FDEs such as those proposed in [3] are not supported. Despite the fact that the examples shown use a single FDE, the subroutine provided can be used for multiple FDEs. However, it is worth mentioning that regarding the multi compartment analysis, one needs to take precautions when using FDEs in PK problems. As discussed in [4], if the fractional derivatives used in each equation have different orders $\alpha _{1}$, $\alpha _2$ etc., i.e. the non-commensurate case, then mass balance issues arise. This happens because usually the outgoing flux from one compartment is an incoming flux in another. If different orders are used, then the transfer rate constants will have different units (i.e. $h^{-a_{1}}$, $h^{-a_2},$ etc.) and mass balance will not hold. Fractional multi-compartmental models can be useful if the same order $\alpha$ is used (commensurate case), in which case the equations are the same as in the classic case, simply by changing the order of the derivatives on the left hand side of the ODEs, or when studying problems where mass balance is not an issue such as PK/PD models, where there is no mass balance between the PK and the PD. This gives plenty of opportunities for future studies on PK/PD data [7]. Pharmacological signals are often characterised by delayed response and the history of previous states of involved variables needs to be taken into account in the models, instead of the instantaneous local state of the variables. FDEs provide this capability, along with other approaches which differ mathematically, but offer a similar flavour, such as delay differential equations, which have found important applications in pharmacometrics [25].

A final interesting comment about the current analysis is that in all cases the order of the FDE, $\alpha$ , has been kept the same for all subjects, without IIV. Indeed it is not clear what could be the physical meaning of IIV on $\alpha$, since this parameter has so fundamental impact on the problem and even affects the units of other parameters. Considering a statistical distribution on a parameter such as $\alpha$ seems to run into theoretical problems even by attempting to define it, despite the fact that from a numerical point of view it could be possible to estimate an OMEGA associated to $\alpha$.

## Conclusions

The present study implements FDE systems in NONMEM with the aim to allow the use of fractional PK models in NONMEM and to the best of our knowledge it is the first attempt to use FDE systems in a NLMEs framework, so the approach could be of interest to other disciplines apart from PKs. The method supports linear and non-linear models with multiple equations and multiple doses. In simulation studies the method proved capable to provide unbiased and precise estimates of the parameter values, while with a real PK data set, performed well, and gave an alternative, similarly good fit to a classic 3-compartment model, but more parsimonious. As a first attempt to introduce FDEs in the NLME framework there are limitations that will be addressed in future work, furthermore it is hoped that the present provided software will motivate more applications.

## Declarations

### Supplementary information

The code used in the article can be found in the GitHub page: https://github.com/PMXathens/FDE4NONMEM
